# Supplementary material for: Understanding financial hardship in families of people living with dementia: Protocol for a scoping review to identify subjective self-report measures that evaluate financial hardship
Source: PLoS One. 2025 Sep 9;20(9):e0331114. doi: 10.1371/journal.pone.0331114 (PMC12419593; doi:10.1371/journal.pone.0331114)
Supplement: S1 Appendix — (DOCX) [file pone.0331114.s001.docx]

**S1 Appendix.** Example Search.

Embase.com search:

('financial stress'/exp OR 'financial security'/de OR 'income security'/de OR 'economic well-being'/de OR 'cost of illness'/de OR 'Caregiver Strain Index'/de OR 'absenteeism'/de OR **'poverty'/de** OR (bankrupt* OR asset-depletion OR depletion-of-asset* OR asset-limit* OR economic-aspect*-of-illness OR **economic-burden-of-disease** OR cost*-of-illness OR illness-cost* OR economic-quality-of-life OR impoverish* OR poverty OR debt* OR indebt* OR ability-to-pay OR caregiver-strain-index OR lack-of-money OR lacking-money OR medical-indigenc* OR medical-debt* OR unpaid-care OR unpaid-caregiving OR absenteeism OR disability-absence OR sickness-absen* OR work-time-loss OR work-absen* OR work-day-loss* OR work-time-loss* OR loss-of-productivity OR productivity-loss* OR work-impairment OR employment-loss OR unpaid OR uncompensated OR without-reimbursement OR unreimbursed OR ((asset* OR savings) **NEAR/4** (depletion OR depleted OR limit*)) OR ((financ* OR income OR money OR economic) **NEAR/4** (stress* OR security OR insecurit* OR crisis OR crises OR challeng* OR hardship* OR pressure* OR strain* OR toxicit* OR burden* OR difficult* OR worr* OR trouble* OR distress* OR impact* OR well-being OR wellbeing OR wellness OR quality-of-life OR life-quality OR qol OR concern* OR need* OR problem* OR debt* OR indebt* OR deficit* OR adequacy OR inadequacy OR anxiet*)) OR ((out-of-pocket OR hidden OR indirect OR intangible OR informal OR catastrophic OR unplanned OR unexpected OR illness) **NEAR/4** (cost OR costs OR expense* OR payment* OR spending OR expenditure*))):ab,ti) **AND** ('self report'/exp OR 'patient-reported outcome'/de OR ‘questionnaire’/exp OR (self-report* OR selfreport* OR patientreport* OR patient-report* OR PROM OR PROMS OR WPAI OR work-productivity-and-impairment OR WLQ OR work-limitations-questionnaire OR ((patient* OR self OR carer* OR caregiver* OR famil* OR stepfamily* OR step-famil* OR blended-famil* OR step-kin OR stepparent* OR step-parent* OR stepchild* OR stepdaughter* OR step-daughter* OR stepson* OR step-son* OR adopted-child* OR foster-child* OR grandparent* OR grandmother* OR grandfather* OR grandchild* OR granddaughter* OR grandson* OR niece* OR nephew* OR sibling* OR sister* OR brother* OR sister-in-law* OR sisters-in-law* OR brother-in-law* OR brothers-in-law* OR aunt* OR uncle* OR cousin* OR ex-wife* OR ex-wives* OR ex-husband* OR family-of-choice* OR families-of-choice* OR spouse* OR domestic-partner* OR partner* OR husband* OR wife* OR sibling* OR child* OR daughter* OR son OR sons OR patient-caregiver* OR caregiver-patient* OR relative*) **NEAR/4** (survey* OR questionnaire* OR scale* OR score* OR scoring OR screen* OR model* OR measur* OR rating* OR long-form* OR short-form* OR assessment* OR report*))):ab,ti) **AND** ('dementia'/de OR 'Alzheimer disease'/de OR 'diffuse Lewy body disease'/de OR 'frontotemporal dementia'/exp OR 'mixed dementia'/de OR 'mixed depression and dementia'/de OR 'multiinfarct dementia'/de OR 'presenile dementia'/de OR 'senile dementia'/de OR (amentia* OR demention* OR dementia* OR alzeimer* OR Alzheimer* OR diffuse-cortical-scleros* OR DLB OR DLBD OR pick*-disease* OR wilhelmsen-lynch-disease* OR ((dementia*) **NEAR/3** (lewy OR front* OR disinhibition)) OR lewy-body-disease* OR fvFTD OR bvFTD OR FTD OR FLTD OR ftd-grn OR hddd1 OR pick*-complex OR senile-confusion OR senile-psychosis OR ((lacunar OR multi-infarct* OR multiinfarct* OR vascular OR presenile OR senile OR senilis) **NEAR/3** (dementia*)) OR ((behavioral OR behavioural OR frontal) **NEAR/3** (FTD))):ab,ti)
