# Supplementary material for: Understanding financial hardship in families of people living with dementia: Protocol for a scoping review to identify subjective self-report measures that evaluate financial hardship
Source: PLoS One. 2025 Sep 9;20(9):e0331114. doi: 10.1371/journal.pone.0331114 (PMC12419593; doi:10.1371/journal.pone.0331114)
Supplement: S2 Appendix — (DOCX) [file pone.0331114.s002.docx]

**S2 Appendix.** Proposed search criteria for AI-based extraction

**“Article title”**

Extract the full title of the article

**“Author Details”**

Extract the following from the first author: last name, first initial

**“Year of publication”**

Extract the year the article was published

**“Sample size”**

Extract the total number of study participants in the article. For articles covering multi-stage studies or multiple sub-studies, report each sample size separately for each sub-study. Report sample sizes for the study populations separately. Include numbers for different study groups such as: caregivers vs. patients, or different caregiver groups

**“Subjective, self-report measures of financial constructs”**

Provide the names of the assessments, measures, item(s) or variables that the caregiver/care partner or family member completed. Only include self-report surveys, measures, items, or variables that the caregiver/care partner or family member completed. Exclude proxy measures that the caregivers/care partners completed. Include only assessments, measurements, or tools which measure financial constructs, such as financial hardship, financial strain, asset depletion, etc. Include measures such as resource utilization or work productivity impairment, but exclude demographic variables or objective financial/SES questions/items. If financial measurements in the article are cost estimates of care based on other known costs of services, state “cost estimate based on services utilized.”

**“Sample-specific reliability”**

Provide any data or information on reliability of the self-report financial constructs, surveys, assessments, or measures from the article. This would include any analysis which refers to the consistency and stability of a measure, meaning that it produces similar results when applied repeatedly. Examples of psychometric reliability testing include internal consistency or test-retest reliability for the financial construct.

**“Sample-specific validity”**

Provide any data or information on validity of the self-report financial constructs, surveys, assessments, or measures from the article. This would include any analysis which refers to the extent to which a test measures what it's supposed to measure. Examples of psychometric validity testing include convergent or discriminant validity, known-groups validity, or responsiveness data for the financial construct.
