# Supplementary material for: Understanding financial hardship in families of people living with dementia: Protocol for a scoping review to identify subjective self-report measures that evaluate financial hardship
Source: PLoS One. 2025 Sep 9;20(9):e0331114. doi: 10.1371/journal.pone.0331114 (PMC12419593; doi:10.1371/journal.pone.0331114)
Supplement: S3 Table — (DOCX) [file pone.0331114.s003.docx]

**S3 Table.** Summary of features to be extracted from text

| Study Identifier | First Author, year | N | Subjective self-report measure | Internal consistency reliability (e.g., Cronbach’s Alpha | Test-retest reliability (*r)* | Convergent validity  (correlations with other, similar constricts) | Discriminant validity (correlation with dissimilar constructs) | Known-groups validity (significant group differences for samples known to be different)  Group means and p value | Responsiveness or change over time data |
| --- | --- | --- | --- | --- | --- | --- | --- | --- | --- |
|  |  |  |  |  |  |  |  |  |  |
|  |  |  |  |  |  |  |  |  |  |
|  |  |  |  |  |  |  |  |  |  |
|  |  |  |  |  |  |  |  |  |  |
