# Supplementary material for: Understanding financial hardship in families of people living with dementia: Protocol for a scoping review to identify subjective self-report measures that evaluate financial hardship
Source: PLoS One. 2025 Sep 9;20(9):e0331114. doi: 10.1371/journal.pone.0331114 (PMC12419593; doi:10.1371/journal.pone.0331114)
Supplement: Attachment — Submitted filename: FISCAL response to reviewer comments 20250725.docx [file pone.0331114.s005.docx]

**Editor’s Comments:**

Editor Comment 1: Please ensure that your manuscript meets PLOS ONE's style requirements, including those for file naming

***Response*: We have confirmed that the submitted manuscript meets all specifications for style and formatting. We have named the resubmitted files as requested.**

Editor Comment 2: Thank you for stating the following financial disclosure:

“Work on this manuscript was supported by grant number R01AG088345(PI: Carlozzi, N.E.) from the National Institutes of Health (NIH), National Institute on Aging. Funders did not play a role in the development of the protocol. Dr. Leggett is also funded by NIAP30AB072931.”

Please state what role the funders took in the study. If the funders had no role, please state: "The funders had no role in study design, data collection and analysis, decision to publish, or preparation of the manuscript."

If this statement is not correct you must amend it as needed.

Please include this amended Role of Funder statement in your cover letter; we will change the online submission form on your behalf.

***Response:* We have added the above statement to the financial disclosure statement in the manuscript (lines 224-228) as well as the cover letter, confirming that the funders had no role in the study or manuscript.**

Editor Comment 3: Please amend either the title on the online submission form (via Edit Submission) or the title in the manuscript so that they are identical.

***Response:* We have amended the title in the manuscript to match the title in the online submission form.**

Editor Comment 4: Please review your reference list to ensure that it is complete and correct. If you have cited papers that have been retracted, please include the rationale for doing so in the manuscript text, or remove these references and replace them with relevant current references. Any changes to the reference list should be mentioned in the rebuttal letter that accompanies your revised manuscript. If you need to cite a retracted article, indicate the article’s retracted status in the References list and also include a citation and full reference for the retraction notice.

***Response:* We have reviewed the reference list, and confirm that it is complete and correct. No cited papers have been retracted, and the only changes to the reference list have been to add additional references in response to specific reviewer comments below.**

-------------------------------------------------------------------------------------------------------------------------------

**Reviewer 1’s Comments:**

Reviewer 1 Comment 1: This protocol is well-structured and follows recognized standards (PRISMA-P/ScR), and the research question is relevant and timely. As this is a protocol paper, the methodological structure is largely fixed, and there is limited value in suggesting design changes at this stage.

***Response*: Thank you for this feedback.**

Reviewer 1 Comment 2: However, as a reader, I would benefit from a clearer conceptual justification of why existing financial hardship instruments developed for cancer and other chronic illnesses are insufficient or inappropriate for use in Alzheimer’s disease and related dementias (ADRD). The authors briefly mention differences in household dynamics and duration of disease, but these important distinctions could be further developed.

Specifically, I encourage the authors to elaborate more explicitly on:

How intergenerational and cross-household financial support systems in ADRD differ from those in cancer care.

Why measures like the COST-FACIT or Economic Strain Model fail to capture critical aspects of financial hardship in ADRD caregiving.

What types of measurement domains might be uniquely required in the context of ADRD.

Expanding this rationale will not only strengthen the justification for the proposed new PRO system but also provide valuable guidance for future users or developers of ADRD-specific instruments.

***Response:* Thank you for these suggestions. We have added additional references and text to the introduction to elaborate on these points (lines 69-95, 102-105).**
